# Gut Microbiome and Metabolome Profiles Associated with High-Fat Diet in Mice

**DOI:** 10.3390/metabo11080482

**Published:** 2021-07-27

**Authors:** Jae-Kwon Jo, Seung-Ho Seo, Seong-Eun Park, Hyun-Woo Kim, Eun-Ju Kim, Jeong-Sang Kim, Ju-Yeon Pyo, Kwang-Moon Cho, Sun-Jae Kwon, Dae-Hun Park, Hong-Seok Son

**Affiliations:** 1 Department of Biotechnology, College of Life Sciences and Biotechnology, Korea University, Seoul 02841, Korea; jojk89@naver.com (J.-K.J.); seong9525@naver.com (S.-E.P.); mn40120@naver.com (H.-W.K.); 2 Sonlab Inc., Seoul 02841, Korea; blue784300@naver.com; 3 College of Korean Medicine, Dongshin University, Naju 58245, Korea; yci3431@naver.com (E.-J.K.); jskim@dsu.ac.kr (J.-S.K.); 4 Department of Pathology, Catholic Kwandong University International St. Mary’s Hospital, Incheon 22711, Korea; jypyo@ish.ac.kr; 5 AccuGene Inc., Incheon 22006, Korea; kmcho@accugenelab.com (K.-M.C.); sjaes@accugenelab.com (S.-J.K.)

**Keywords:** obesity, gut microbiota, metabolite, high-fat diet

## Abstract

Obesity can be caused by microbes producing metabolites; it is thus important to determine the correlation between gut microbes and metabolites. This study aimed to identify gut microbiota-metabolomic signatures that change with a high-fat diet and understand the underlying mechanisms. To investigate the profiles of the gut microbiota and metabolites that changed after a 60% fat diet for 8 weeks, 16S rRNA gene amplicon sequencing and gas chromatography-mass spectrometry (GC-MS)-based metabolomic analyses were performed. Mice belonging to the HFD group showed a significant decrease in the relative abundance of Bacteroidetes but an increase in the relative abundance of Firmicutes compared to the control group. The relative abundance of Firmicutes, such as *Lactococcus, Blautia, Lachnoclostridium, Oscillibacter, Ruminiclostridium, Harryflintia, Lactobacillus, Oscillospira*, and *Erysipelatoclostridium*, was significantly higher in the HFD group than in the control group. The increased relative abundance of Firmicutes in the HFD group was positively correlated with fecal ribose, hypoxanthine, fructose, glycolic acid, ornithine, serum inositol, tyrosine, and glycine. Metabolic pathways affected by a high fat diet on serum were involved in aminoacyl-tRNA biosynthesis, glycine, serine and threonine metabolism, cysteine and methionine metabolism, glyoxylate and dicarboxylate metabolism, and phenylalanine, tyrosine, and trypto-phan biosynthesis. This study provides insight into the dysbiosis of gut microbiota and metabolites altered by HFD and may help to understand the mechanisms underlying obesity mediated by gut microbiota.

## 1. Introduction

Obesity is defined as the excessive accumulation of fat and it results from an imbalance between energy intake and expenditure [1,2]. It is associated with substantial health risks, such as type 2 diabetes, strokes, arthritis, and several types of cancers [3]. Over the past few decades, there has been a remarkable increase in obesity amongst individuals of all ages across the globe. Moreover, no single factor successfully explains the cause of this obesity epidemic [4]. It is well known that obesity is a complex multifactorial disease that is influenced by genetic and environmental factors [5].

Many resident bacteria adapt to the gut environment and develop complex interactions with other bacteria. Gut bacterial communities have many different forms of coexistence, such as constant competition, mutualism, and antagonism [6]. Gut microbiota has drawn considerable attention from the scientific community over the last decade because of its ability to directly affect the health or disease status of an individual. Obesity-related changes in gut bacteria may contribute to weight gain and inflammation, which has been thought to be one of the hallmarks of HFD-induced obesity or obesity-related diseases. For example, gut microbiota can influence the effector molecules that determine fat storage in adipocytes, thus affecting host nutrients acquisition and energy homeostasis [7]. In particular, the community structure of gut bacteria has been implicated in the etiology of obesity in animal studies. For example, the diversity of bacteria differed between the lean and obese mice groups, with obese mice group showing an increase in the Firmicutes/Bacterioidetes ratio [8,9,10,11]. However, in a human translation of this result, association between the Firmicutes/Bacterioidetes ratio and obesity has been controversial [12].

To understand the complex association between human and microbial ecosystems, it is important to adopt comprehensive analytical approaches that capture the dynamic interactions between diet, microbiota, and the human host [13]. In this context, combining the different omic sciences has received considerable interest in obesity research. Metabolomics can help define the metabolites (qualitatively and quantitatively) that are involved in host-microbe interactions, such as those originating from the bacterial conversion of nutrients and host metabolites in the gut lumen that are subsequently transported throughout the body [14]. High-throughput analytical platforms, such as gas chromatography mass spectrometry (GC-MS), have recently been used to improve our understanding of disease processes [15], biomarker discovery [16], and especially, microbiome-host interactions [17,18]. A number of metagenomic and metabolomic approaches are being developed to characterize the phenotype of obese individuals and to represent the crucial metabolic processes that govern the human-bacteria interplay [19]. However, the correlation between gut microbiota and metabolites remains to be fully understood in the context of obesity.

In the present study, 16S rRNA gene amplicon sequencing and GC-MS-based metabolomics were applied to understand the different gut microbiota and metabolite profiles from the serum and fecal sample of the high-fat diet (HFD) and control groups. Understanding the changes in the microbiota and metabolites of HFD-induced obese mice will help in the diagnosis of biomarkers and treatment of obesity.

## 2. Results

### 2.1. Body Weight and Fat Accumulation

**Figure 1:**
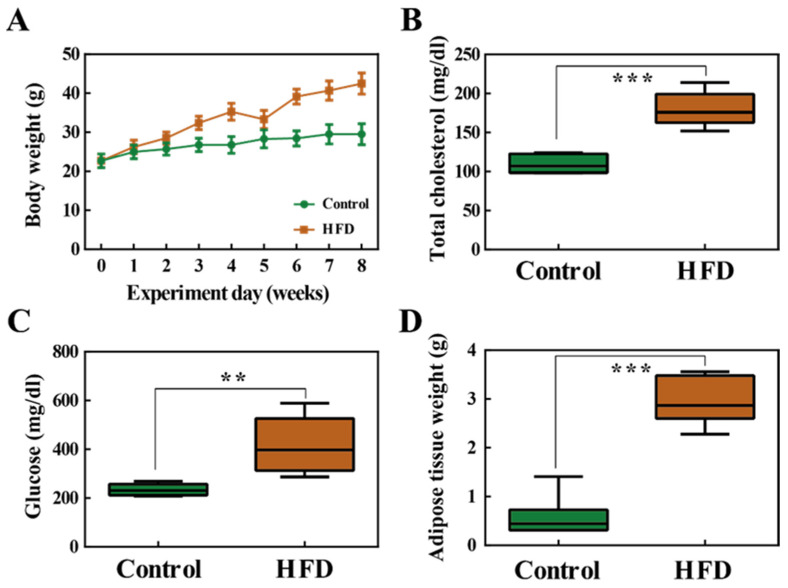
(**A**) Body weight changes in the control group and high fat diet (HFD) group. (**B**) Total cholesterol (TCHO-P III) in serum samples. (**C**) Serum glucose (GLU-P III) levels. (**D**) Weight of the adipose tissue. **, *p* < 0.01; ***, *p* < 0.001.

By the end of the 8 week high-fat diet (HFD) feeding period, the average body weight of the HFD group significantly increased compared to that of the control group (Figure 1A). The levels of total cholesterol (TCHO-P III) and glucose (GLU-P III) in the serum were also significantly increased in the HFD group (Figure 1B,C). There was also a significant increase in adipose tissue weight in the HFD group (Figure 1D). In conclusion, these results show that 8 weeks of HFD induced obesity.

### 2.2. The Composition of the Gut Microbiota

**Figure 2:**
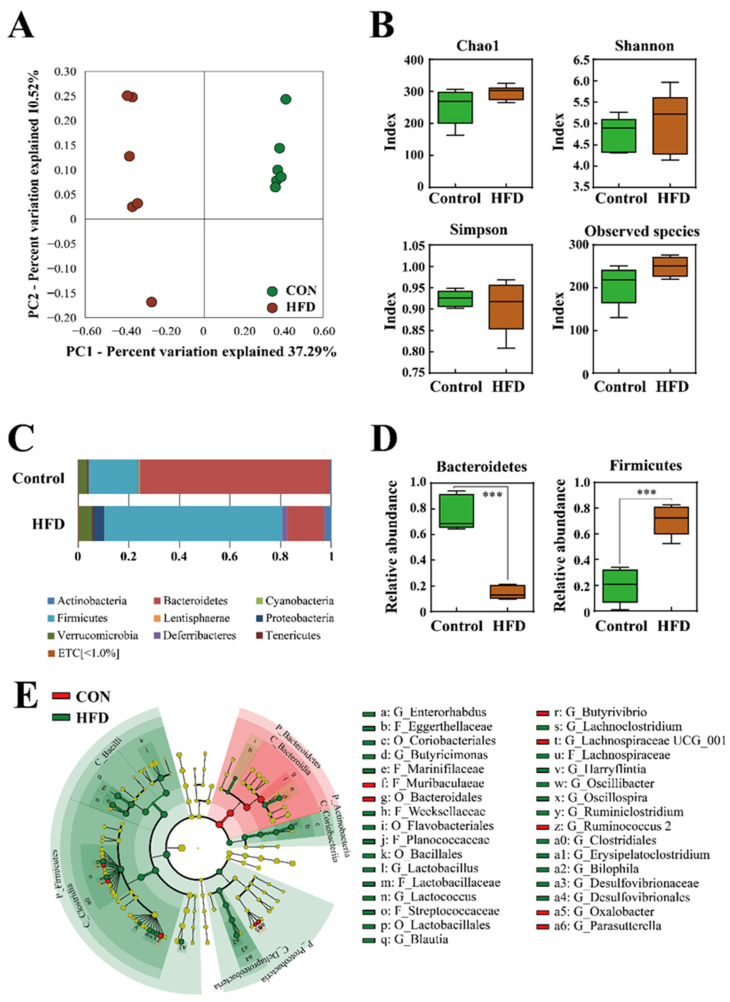
(**A**) Beta diversity analysis of the control and HFD groups. (**B**) Alpha diversity analysis of the control and HFD groups. (**C**) Comparison of microbiota composition at the phylum level. (**D**) Relative abundance of Bacteroidetes and Firmicutes. (**E**) Cladogram, generated using the linear discriminant analysis effect size (LEfSe) method, shows the phylogenetic distribution of microbes that are associated with the control and HFD groups. Taxonomic levels of phylum, class, and order are labelled, while family and genus are abbreviated. Plots were represented using LEfSe. Colored regions/branches indicate differences in the bacterial population structure between the control and HFD groups. Regions in green indicate clades that were enriched in the HFD group compared to those in the control group, while regions in red indicate clades that were enriched in the control group as opposed to those in the HFD group. ***, *p* < 0.001.

To examine whether HFD affects the gut microbiota, bacterial 16S rRNA gene sequencing of fecal samples was performed. A total of 1,171,031 sequences were obtained from 12 fecal samples. The number of reads per sample ranged from 13,908 to 191,289. The number of sequences per sample was normalized based on the minimum number of reads and used for the analysis. The beta diversity was assessed by principal coordinate analysis (PCoA) on weighted Bray-Curtis distance matrices. PCoA revealed that HFD influenced the composition of the gut microbiota (PC1, 37.29%) (Figure 2A). Obesity is known to reduce bacterial diversity and richness [12,20]. However, in the present study, the alpha diversity indices for the observed species, Shannon, Simpson, and Chao1 (richness and evenness), did not differ significantly between the control or HFD groups (Figure 2B).

At the phylum level, the HFD group showed a significant decrease in the relative abundance of Bacteroidetes but an increase in that of Firmicutes, compared to the control group (Figure 2C,D) (*p* < 0.001). Obesity has been associated with a low ratio of Bacteroidetes to Firmicutes, which is consistent with the results of the present study. The differentially abundant taxa were further confirmed by linear discriminant analysis effect size (LEfSe), which exploits linear discriminant analysis (LDA) to identify features that are statistically different among classes [21]. Figure 2E shows the most relevant clades identified by LEfSe (LDA score > 3.0). The resulting cladogram revealed that *Muribaculaceae, Butyrivibrio, Lachnospiraceae* UCG_001, *Rumicoccus*, and *Oxalobacter* were more dominant in the control group than in the HFD group, whereas the HFD group was enriched with *Enterohabdus*, *Eggerthellaceae*, *Coriobacteriales*, *Butyricinomas*, *Oscillibacter*, and *Ruminiclostridium*.

**Figure 3:**
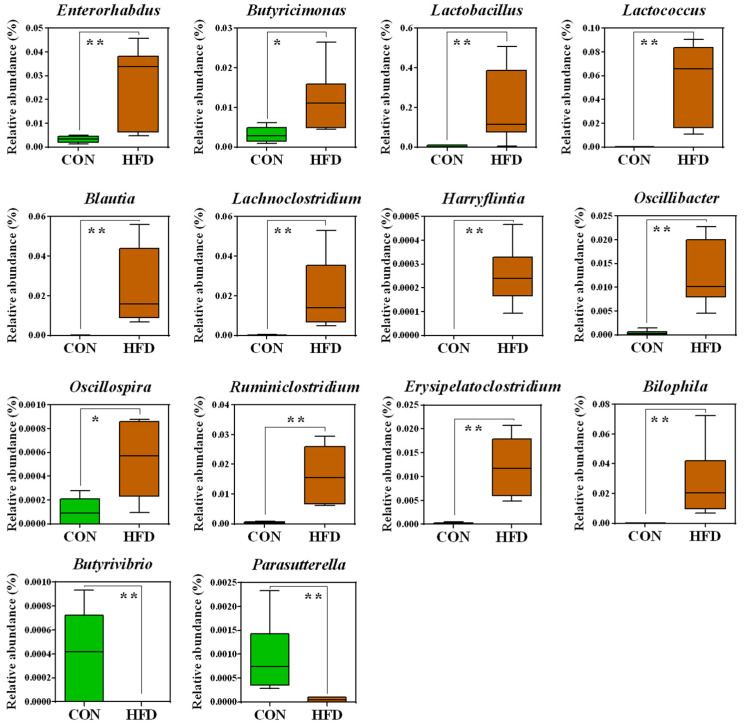
Box plots of significantly different microorganisms at the genus level in the guts of the HFD and control groups. The *p*-values were obtained using Mann–Whitney U tests. *, *p* < 0.05; **, *p* < 0.01. A false discovery rate of 5% was applied to all tests to correct for multiple testing.

Significant bacterial differences at the genus level in the gut bacteria of the HFD and control groups are shown in Figure 3. The relative abundances of 12 taxa, i.e., *Enterorhabdus* (*p* < 0.01), *Butyricimonas* (*p* < 0.05), *Lactococcus* (*p* < 0.01), *Blautia* (*p* < 0.01), *Lachnoclostridium* (*p* < 0.01), *Oscillibacter* (*p* < 0.01), *Ruminiclostridium* (*p* < 0.01), *Harryflintia* (*p* < 0.01), *Lactobacillus* (*p* < 0.01), *Oscillospira* (*p* < 0.05), *Erysipelatoclostridium* (*p* < 0.01), and *Bilophila* (*p* < 0.01), were significantly higher in the HFD group than in the control group. Conversely, the control group had significantly higher relative abundances of *Butyrivibrio* (*p* < 0.01) and *Parasutterella* (*p* < 0.01).

To investigate the differences in microbial functions between the control group and HFD group, based on the KEGG database, we adopted PICRUSt using 16S rRNA gene profiles (Figure S1 and Tables S1 and S2).

### 2.3. Serum and Feces Metabolites

**Figure 4:**
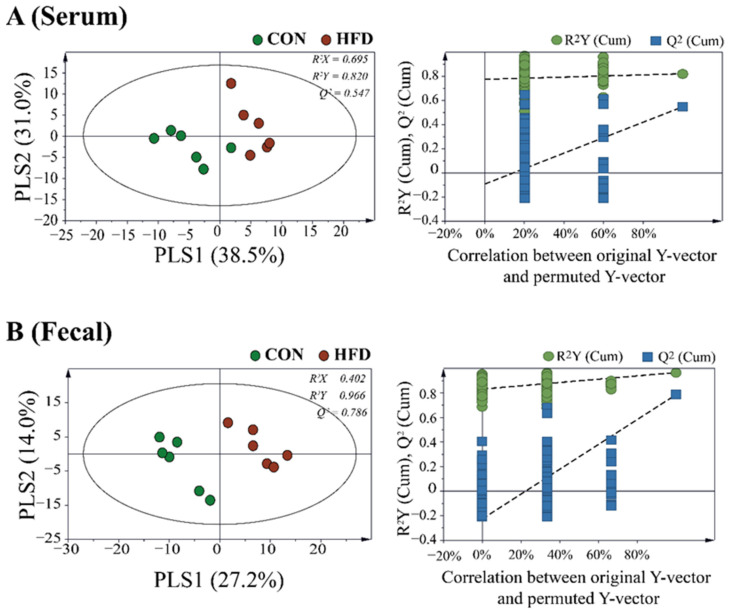
Supervised partial least squares discriminant analysis (PLS−DA) score plot derived from the GC−MS data of (**A**) serum and (**B**) fecal samples of HFD and control groups. Permutation tests with 200 iterations were performed to validate the goodness of fit of the original model.

To study the different metabolite profiles in the serum and feces of the HFD and control groups, partial least squares discriminant analysis (PLS-DA) was applied to GC-MS data (Figure 4). The PLS-DA model of serum and fecal samples showed clear separation between the control and HFD groups, indicating that the metabolic profiles of the HFD group were different from those of the control group.

**Figure 5:**
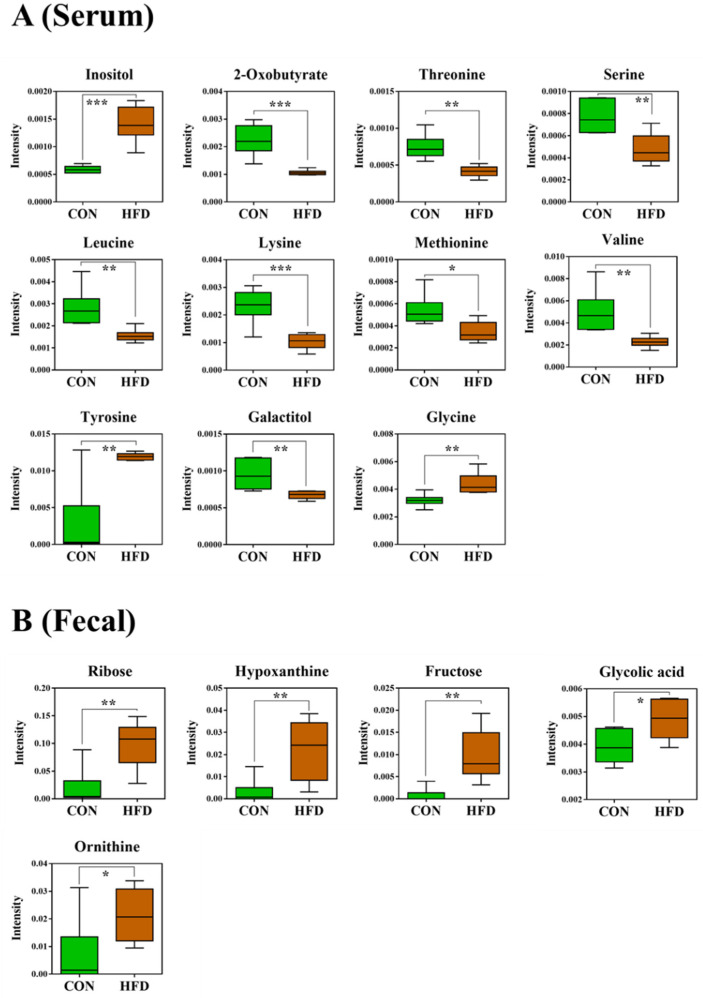
Box plots of significantly different metabolites in (**A**) serum and (**B**) fecal samples of the HFD and control groups. *, *p* < 0.05; **, *p* < 0.01; ***, *p* < 0.001. A false discovery rate of 5% was applied to all tests to correct for multiple testing.

Among the 127 metabolites that were detected in the serum, those that significantly contributed to clustering between the control and HFD groups were identified according to a threshold of variable importance in projection (VIP) > 1.0, *p* < 0.05. Figure 5A shows the relative differences between the metabolites identified from the serum and fecal samples of the control and HFD groups. Serum from the HFD group showed high levels of inositol (*p* < 0.001), tyrosine (*p* < 0.01), and glycine (*p* < 0.01). In contrast, the HFD group had significantly lower levels of 2-oxobutyrate (*p* < 0.001), threonine (*p* < 0.01), serine (*p* < 0.01), leucine (*p* < 0.01), lysine (*p* < 0.001), methionine (*p* < 0.05), valine (*p* < 0.01), and galactitol (*p* < 0.01) compared to the control group (Figure 5A). In the present study, the serum levels of lysine and methionine were negatively correlated with obesity, while inositol and tyrosine were positively correlated with obesity. The results for lysine and methionine were similar to the results from previous studies [22,23]. Similarly, He et al. [24] reported that the serum of obese individuals had high levels of inositol and tyrosine using NMR-based metabolomic technology, which is consistent with our results for inositol and tyrosine.

Among the 355 metabolites detected in fecal samples, those that significantly contributed to the discrimination were identified according to a threshold of VIP > 1.0, *p* < 0.05. The levels of ribose (*p* < 0.01), hypoxanthine (*p* < 0.01), fructose (*p* < 0.01), glycolic acid (*p* < 0.05), and ornithine (*p* < 0.05) were significantly higher in the HFD group than in the control group (Figure 5B). Ribose is known to affect gut activity [25,26]. However, many studies have shown variations in the levels of fecal ribose between HFD and control groups [27,28]. The HFD group had higher levels of hypoxanthine than the control group, in contrast to other studies [29].

### 2.4. Metabolic Pathway Analysis

Metabolic pathway analysis was performed to identify relevant metabolic pathways affected by a high fat diet. This analysis shows metabolic pathways by enrichment analysis and impact values by topology analysis. Important pathways were identified, based on the pathway impact and −log (*p*) value. Metabolic pathways affected by a high fat diet on serum were involved in aminoacyl-tRNA biosynthesis, glycine, serine, and threonine metabolism, cysteine and methionine metabolism, glyoxylate and dicarboxylate metabolism, and phenylalanine, tyrosine, and tryptophan biosynthesis (Table S3). Figure S2 shows a schematic of the affected metabolic pathways by a high fat diet on serum. Metabolic pathways affected by a high fat diet on feces were involved in glutathione metabolism, phenylalanine, and tyrosine and tryptophan biosynthesis (Table S4). Figure S3 shows a schematic of the affected metabolic pathways by a high fat diet on feces.

### 2.5. Correlation between Microbiota and Metabolites

To understand the relationships between gut microbiota and the metabolites in serum and feces, Pearson correlations were used to generate a correlation matrix (|r| > 0.7). Fructose levels in feces were positively correlated with the relative abundance of *Enterorhabdus* (r = 0.702), *Lactococcus* (r = 0.774), *Lachnoclostridium* (r = 0.766), *Oscillibacter* (r = 0.753), and *Ruminiclostridium* (r = 0.707) that belong to the phylum Firmicutes. Glycolic acid levels in feces correlated positively with the relative abundance of *Lactobacillus* (r = 0.724). Serum inositol levels correlated positively with the relative abundance of *Butyricimonas* (r = 0.701), *Harryflintia* (r = 0.766), *Oscillibacter* (r = 0.733), and *Ruminiclostridium* (r = 0.793). Serum glycine levels correlated positively with the relative abundance of *Lactobacillus* (r = 0.722). Serum 2-oxobutyrate levels correlated negatively with the relative abundance of *Harryflintia* (r = −0.712) and *Oscillibacter* (r = −0.710).

**Figure 6:**
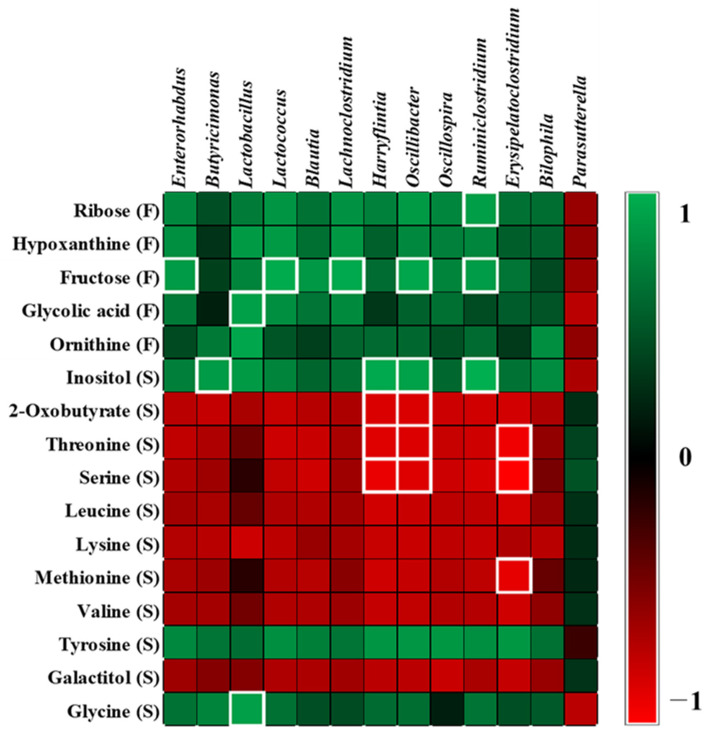
The heat map shows correlation between the identified metabolites and microbiota. R−values of 0.7 or more are highlighted with white borders. A false discovery rate of 5% was applied to all tests to correct for multiple testing.

Serum threonine levels correlated negatively with the relative abundance of *Harryflintia* (r = −0.727), *Oscillibacter* (r = −0.714), and *Erysipelatoclostridium* (r = −0.786). Serum serine levels correlated negatively with the relative abundance of *Harryflintia* (r = −0.764), *Oscillibacter* (r = −0.724), and *Erysipelatoclostridium* (r = −0.833). Serum methionine levels correlated negatively with *Erysipelatoclostridium* (r = −0.749) (Figure 6).

## 3. Discussion

In previous studies of associations between diet and obesity, it was found that body-weight gain was significantly associated with the amount of fat in the diet rather than protein or carbohydrate. In this study, a 60% fat diet (soybean oil) was used, which is the most used in animal obesity studies. De Wit et al. [30] reported that the quality of dietary fat affects the gut microbiota composition. In their study, only the saturated fats (palm oil diet) altered the microbial diversity and increased the Firmicutes to Bacteroidetes ratio, whereas unsaturated fats (olive oil or safflower oil) did not affect microbial diversity. In addition, recent studies reported that unsaturated fats upregulated specific bacteria (*Lachnospira*, *Roseburia*, and unclassified *Ruminococcaceae*) associated with positive metabolic health and leanness, whereas saturated fats had a positive effect on the abundance of *Bilophila*, which has a genotoxicity [31,32]. These results suggested that the quality and the amounts of fat, were associated with gut microbiota composition. High-fat diet-induced obesity is known to be associated with a low ratio of Bacteroidetes to Firmicutes; this is consistent with the results of the present study. However, some recent studies have reported controversial results, indicating that weight loss did not change the ratio of Firmicutes to Bacteroidetes, and suggest that the Firmicutes to Bacteroidetes ratio is not important for obesity [33,34]. Alternatively, Gupta et al. [35] proposed a new index (Gut Microbiome Health Index) that compares the relative abundances of two sets of microbial species associated with good and adverse health conditions. Therefore, the difference between obese and lean states can be explained at the species level of the gut microbiota. In the present study, the relative abundance of Firmicutes, such as *Lactococcus*, *Blautia*, *Lachnoclostridium*, *Oscillibacter*, *Ruminiclostridium*, *Harryflintia*, *Lactobacillus*, *Oscillospira*, and *Erysipelatoclostridium* was significantly higher in the HFD group than in the control group. These bacteria are known to be significantly associated with inflammation-mediated obesity [36]. Gonzalez-Quintel et al. [37] reported that increased levels of circulating lipopolysaccharide-binding protein were observed in obese individuals. Lipopolysaccharides derived from gram-negative bacteria are thought to generate chronic low-grade inflammation. Subsequently, inflammation is associated with the onset of obesity or obesity-related diseases, such as insulin resistance [38].

In the present study, *Lactobacillus, Blautia, Bilophila*, *Enterorhabdus*, *Oscillibacter,* and *Erysipelatoclostridium* were significantly higher in the HFD group than in the control group. Ignacio et al. [39] demonstrated that *Lactobacillus* was more abundant in obese and overweight children than in lean children and that it correlated positively with body mass index (BMI). Many studies have reported that *Blautia* is positively correlated with obesity [40,41,42]. Hu et al. [41] reported that, during the inhibition of obesity using long-chain bases from sea cucumber, the abundance of *Blautia* and *Enterorhabdus,* which belong to gram-negative bacteria, decreased. The author also showed that these changes were accompanied by a decrease in the lipopolysaccharide level, which is associated with inflammation. Similarly, *Oscillibacter* is known to have a positive correlation with obesity and increased permeability of the mouse colon [43]. The increasing relative abundance of clusters containing *Bilophila* and *Erysipelatoclostridium* has also been positively associated with HFD and inflammation due to progressing obesity [44,45,46].

Branched-chain amino acids (BCAAs), such as valine and leucine, are associated with obesity [47,48]. Considering that BCAAs are essential amino acids for animals and can only be obtained from dietary intake or bacterial metabolism [49], gut microbiota may be important for the supply of BCAAs, such as leucine and valine, to the hosts [50]. Kim et al. [51] reported that, in humans, plasma levels of valine and leucine were higher in overweight/obese males than in lean controls. Newgard et al. [52] reported that important enzymes of the catabolic pathway for BCAAs had lower activities in the adipose tissue of obese mice, leading to an increase in BCAA levels [53]. However, other metabolomic studies have reported a decrease in the serum levels of BCAAs in HFD-induced obese mice [22,54], which is consistent with our results of lower levels of valine and leucine in the HFD group. Lysine acetylation might be an important factor that affects obesity by regulating energy homeostasis [55]. Zhang et al. [56] reported that most acetylphosphate-generating enzymes are derived from Firmicutes that have higher lysine acetylome-to-metaproteome ratios than other bacterial phyla. Threonine levels were higher in the serum from the control group. In addition, metabolic pathway analysis revealed that glycine, serine, and threonine metabolism is affected by high fat diets. Ma et al. [57] reported that dietary supplementation with threonine not only led to a significant decrease in the overall body weight but also in that of the epididymal and perirenal fat pads, suggesting that threonine might be negatively associated with obesity. In the present study, glycolic acid levels were higher in the fecal sample from the HFD group and high fat diet affected glyoxylate and dicarboxylate metabolism, as shown in the results of metabolic pathway analysis. Glycolic acid is involved in glyoxylate and dicarboxylate metabolism and is reported as one of the most probable metabolic pathways used for the classification of obesity-related diseases [58]. Glyoxylate and dicarboxylate cycles are related to energy metabolism [59]. Glycolic acid is a precursor of oxalate. Taylor et al. [60] reported that the levels of oxalate correlated positively with obesity. In the present study, the levels of glycine were higher in the HFD group than in the control group and high fat diets affected the glycine, serine, and threonine metabolism in metabolic pathway analysis. Creatine in muscle is biosynthesized from glycine and broken down to endogenous creatinine [61]. Previous studies have reported increased urinary excretion of creatinine in obese participants and HFD-fed obese mice, presumed to be the result of skeletal and cardiac muscle hypertrophy to support and move the increased body mass [62]. Many studies suggest that creatine metabolism is associated with obesity [63,64]. Kazak et al. [64] reported that inactivated glycine amidinotransferase, an enzyme involved in creatine biosynthesis, makes mice prone to diet-induced obesity due to the suppression of elevated energy expenditure, which occurs upon high-calorie feeding. In the present study, *Lactobacillus* was positively correlated with the relative abundance of glycine (r = 0.722) and glycolic acid (r = 0.724). However, little is known about the relationship between gut bacteria and metabolites.

It is important to explore the correlation between gut microbes and metabolites because obesity can be caused by microbes that produce many metabolites. This study provides insights into the changes induced by HFD in gut microbiota and metabolites. However, HFD feeding for 8 weeks did not reduce alpha diversity in the present study. Additionally, our results for some microbiota and metabolites were not in agreement with those of previous studies on obesity. This might have been due to the small number of experimental animals or due to unclear patterns of changes in the gut microbiota or metabolites caused by obesity. Further studies are needed to ascertain the effects of obesity on the gut microbiota and metabolites.

## 4. Materials and Methods

### 4.1. Animals and Treatment

The experiments were approved by the Animal Ethics Committee of the Dongshin University (Approval No. 2019-07-02). To evaluate the effects of obesity, C57BL/6 mice were purchased from SamTako BioKorea (Osan, Korea) and acclimatized for 7 days. According to the type of diet, 12 mice were divided into two groups (Research Diet, Inc. Product #D12492, New Brunswick, NJ, USA) and given tap water for 8 weeks: (1) control group (*n* = 6); (2) obesity group with a HFD (D12492 Rodent Diet with 60% fat, 20% protein, 20% carbohydrate, Research Diets, Inc., New Brunswick, NY, USA) (*n* = 6). Ingredients used for the control and obesity group diets are provided in Tables S5 and S6.

The body weights of the mice were recorded once per week. All mice were anesthetized with 50 mg/kg Zoletil (Virbac, TX, USA). To determine metabolites in the serum, the collected whole blood was centrifuged at 12,000 rpm using a Legend micro 17-R (Thermo Fisher Scientific, Waltham, MA, USA). TCHO-P III and GLU-P III levels were measured. Mice were euthanized using Zoletil over-dosing, then the abdominal fat tissues were collected and the weights of these tissues were measured.

### 4.2. DNA Extraction and 16S rRNA Gene Amplicon Sequencing

Fecal samples were collected from laboratory mice 8 weeks after they were administered a 60% fat diet. In total, 500 μL of methanol was added to each 100 mg of fecal sample and then vigorously extracted. Fecal DNA was extracted using the AccuFAST automation system (AccuGene, Inc., Incheon, Korea), according to the manufacturer’s instructions. For MiSeq sequencing, bacterial genomic DNA amplification was performed using primers containing 515fb and 806rb and Nextera adaptor sequences to target the V4 hypervariable region of the 16S rRNA genes [65]. The 16S rRNA genes were amplified using 25 polymerase chain reaction (PCR) cycles and KAPA HiFi HotStart ReadyMix (Roche sequencing, Pleasanton, CA, USA). The PCR products (~250 bp) were purified using HiAccuBeads (AccuGene, Inc., Incheon, Korea). Amplicon libraries were pooled at equimolar ratios. The pooled libraries were sequenced on the Illumina MiSeq system using the MiSeq Reagent Kit v2 for 500 cycles (Illumina, San Diego, CA, USA). The raw sequencing reads for all raw data sets were subjected to reference-based chimera filtering using VSEARCH v2.10.3 [66]. The chimeric filtered sequences were assigned to operational taxonomic units (OTUs) by OTU picking using the QIIME pipeline (http://www.qiime.org/, accessed on 1 May 2021). Sequences were clustered using UCLUST into OTUs based on the SILVA 132 (pre-clustered at 97% similarity threshold) database.

### 4.3. Sample Derivatization and GC-MS Analysis

A total of 200 μL of cold methanol was added to 20 μL of serum or a freeze dried fecal (50 mg) sample and then vigorously extracted. After centrifugation at 12,000 rpm, 5 min, at 4 °C, 100 μL of the supernatant was freeze-dried. Equal volumes of each sample were collected and prepared for quality control analysis. The methods of sample derivatization and GC-MS analysis have been described previously [67]. Briefly, 80 µL of O-methoxyamine hydrochloride (20 mg/mL) was added to each freeze-dried sample and incubated (75 rpm, 30 °C, 90 min) in the dark. Silylation was performed by adding 30 µL of N-methyl-N-trimethylsilyl-trifluoroacetamide. Each sample was vortex mixed, shaken (75 rpm), and incubated at 37 °C for 30 min. After centrifuging the sample at 13,000 rpm for 10 min, GC-MS analysis was performed on the supernatant. The derivatized samples were analyzed using a QP 2020 GC-MS (Shimadzu, Kyoto, Japan). The GC oven temperature was initially held at 80 °C for 2 min and finally increased to 330 °C at a rate of 15 °C/min and held for 6 min. The m/z range was set to 85–500, with electron impact ionization (70 eV). A GC solution (Shimadzu, Kyoto, Japan) was employed to obtain chromatograms and mass spectra. The data were converted into a netCDF file after GC-MS analysis and processed for peak detection and alignment using the MetAlign software. MetAlign parameters were set according to the AIoutput scaling requirements: a peak slope factor of 2, peak threshold of 10, average peak width at half height of 25, and peak threshold factor of 4. The resulting data (CSV-format file) were imported into the AIoutput software for peak identification and prediction.

### 4.4. Data Processing and Statistical Analysis

The GC-MS data was analyzed with the help of principal component analysis (PCA) and PLS-DA to visualize the variance of metabolites and using SIMCA-P 15.0 (Umetrics, Umea, Sweden). For model validation, a 200-fold cross validation was performed. Metabolites with a VIP > 1.0, and a *p* < 0.05 were considered different across the two groups. The mass spectra data of the metabolites were compared with the help of the AIoutput software, NIST 14.0 library, and the human metabolome database (HMDB, http://www.hmdb.ca, accessed on 1 May 2021). Metabolic differences between groups were examined for statistical significance using Student’s *t*-test. To determine statistically significant differences between the two groups in microbial analysis, the non-parametric Mann-Whitney U test was used for unpaired data. The Benjamini-Hochberg algorithm was used to control the false discovery rate [68]. A FDA of 5% was applied to all tests to correct for multiple testing. Predictive functional analysis OTU picking from the 16S amplicon sequencing data was performed using QIIM [69] and the greengenes database [70], and functional analysis was conducted on the OTU data using PICRUSt [71]. The predictive functional analysis results were annotated with the Kyoto Encyclopedia of Genes and Genomes (KEGG) pathway [72].

### 4.5. Metabolic Pathway Analysis

Metabolic pathway analysis was performed with the MetaboAnalyst web software (metaboanalyst.ca) and the KEGG by filtering the dataset using the FDR-adjusted *p* value < 0.05 and impact value > 0.1 to reveal how significant metabolites are involved in different pathways [73].

### 4.6. Correlation Analysis

Associations between metabolites and microbes were assessed using Pearson’s correlation analysis. A FDR of 5% was applied to all tests to correct for multiple testing.

## 5. Conclusions

In the present study, HFD feeding had a remarkable effect on changes in the gut bacterial ecosystem, which can adversely affect obesity. The increased relative abundance of Firmicutes in the HFD group showed a positive correlation with some metabolites, suggesting that it may play a key role in the development of obesity in response to a HFD. This study provides insight into the dysbiosis of the gut microbiota and metabolites altered by a HFD and may help to understand the mechanisms underlying obesity mediated by gut microbiota.

## Data Availability

The data presented in this study are available on request from the corresponding author.

## Supplementary Materials

The following are available online at https://www.mdpi.com/article/10.3390/metabo11080482/s1, Figure S1: Distribution of level 1 KEGG functional categories from the CON (green) and HFD (red) groups, Figure S2: Summary of pathway analysis (serum), Figure S3: Summary of pathway analysis (fecal sample), Table S1: The secondary function of the predicted gene, Table S2: The tertiary function of predicted gene, Table S3: Metabolic pathways affected by a high fat diet on serum, Table S4: Metabolic pathways affected by a high fat diet on fecal, Table S5: Diet ingredients of the control group, Table S6: Diet ingredients of the high fat diet group.
