# Supplementary material for: Gut Microbiome and Metabolome Profiles Associated with High-Fat Diet in Mice
Source: Metabolites. 2021 Jul 27;11(8):482. doi: 10.3390/metabo11080482 (PMC8398001; doi:10.3390/metabo11080482)
Supplement: Supplementary file 1 [file metabolites-11-00482-s001.zip › metabolites-1275086-supplementary.pdf]

**Supplementary Materials.**

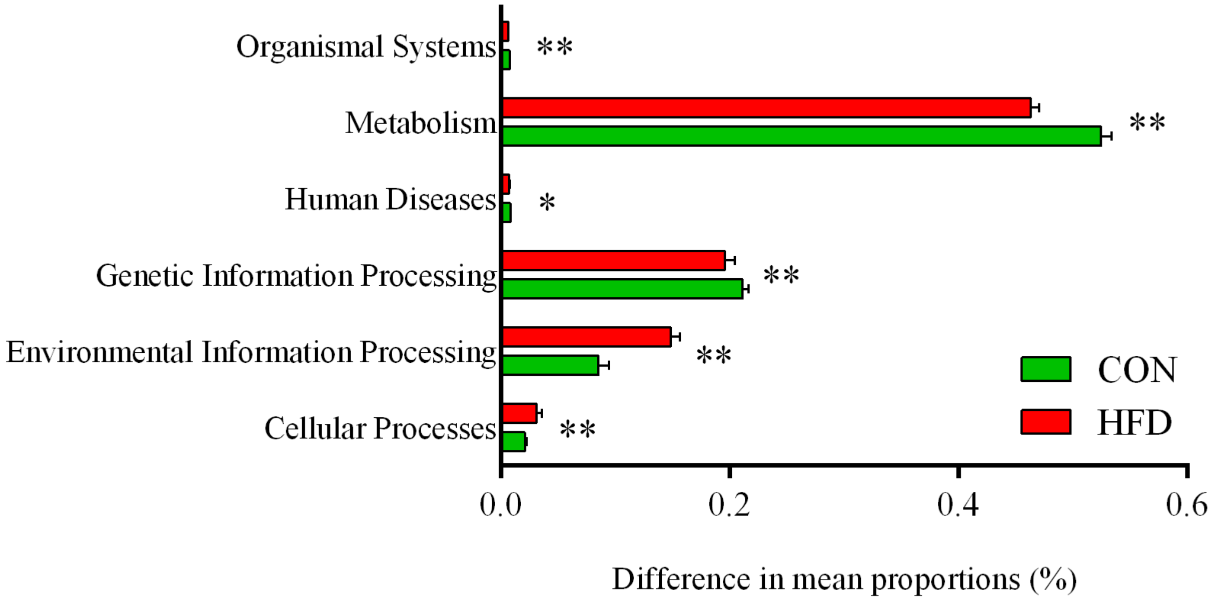


**Figure S1.** Distribution of level 1 KEGG functional categories from the CON (green) and HFD (red) groups.

To investigate the differences in microbial functions between the control group and HFD group, we adopted PICRUSt. Based on KEGG database, PICRUSt revealed a total of six biological metabolism pathways at Level 1 pathways: cellular processes, environmental information processing, genetic information processing, human diseases, metabolism, and organismal systems (Figure S1). Among them, metabolism, genetic information processing and environmental information processing dominated, accounting for 45.38–51.39%, 18.63–21.61% and 7.32–15.76%, respectively. These four pathways (metabolism, genetic information processing, human diseases, and organismal systems) in the HFD group were lower than those in the CON group. Meanwhile, the secondary function of the predicted gene was analyzed, finding it consisted of 24 sub-functions including amino acid metabolism, carbohydrate metabolism, replication and repair, membrane transport (Table S1). In further analysis, we identified 183 high microbial functions at category level 3 of the CON group ($p$< 0.05). In addition, 175 microbial functions were lower in the HFD group ($p$ < 0.05) (Table S2).

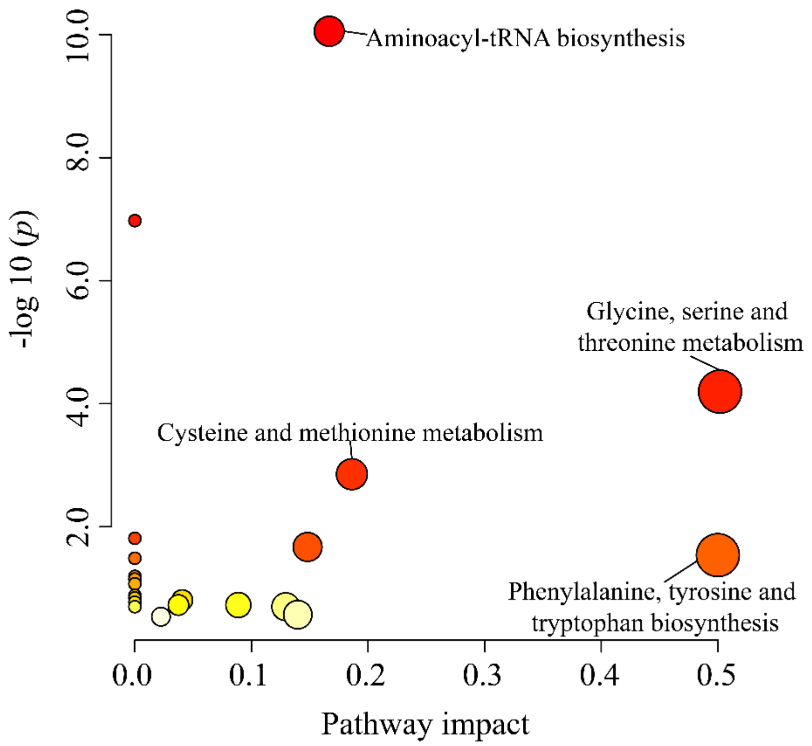


**Figure S2.** Summary of pathway analysis (serum).

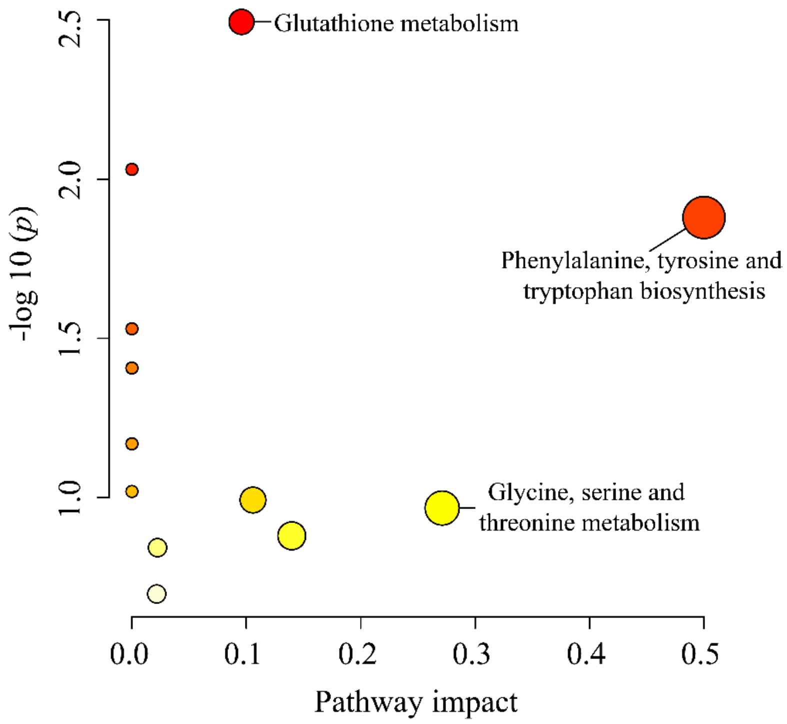


**Figure S3.** Summary of pathway analysis (fecal).

**Table S3.** Metabolic pathways affected by high fat diet on serum.

| No. | Metabolic pathways | Total | Hits | –log($p$) | Impact |
|---|---|---|---|---|---|
| 1 | Aminoacyl-tRNA biosynthesis | 48 | 8 | 10.05 | 0.17 |
| 2 | Glycine, serine and threonine metabolism | 34 | 4 | 4.20 | 0.50 |
| 3 | Cysteine and methionine metabolism | 33 | 3 | 2.85 | 0.19 |
| 4 | Glyoxylate and dicarboxylate metabolism | 32 | 2 | 1.67 | 0.15 |
| 5 | Phenylalanine, tyrosine and tryptophan biosynthesis | 4 | 1 | 1.54 | 0.50 |

**Table S4.** Metabolic pathways affected by high fat diet on fecal.

| No. | Metabolic pathways | Total | Hits | −log(*p*) | Impact |
|---|---|---|---|---|---|
| 1 | Glutathione metabolism | 28 | 2 | 2.49 | 0.10 |
| 2 | Phenylalanine, tyrosine and tryptophan biosynthesis | 4 | 1 | 1.88 | 0.50 |

**Table S5.** Diet ingredients of control group.

사조동아원
SAJO

873, Deokpyeongro, Sunsung-myun,
Dangjin-gun, Choongnam, 31759, Korea
Tel(041)350-6359 Fax(041)350-6376

# ANALYSIS RESULT

**REPORTED March 25, 2021**

**Manufactured by : SajoDongAOne Corporation Ltd**
**USE : LAB Rodent Chow (steam autoclave) Sam #31**
**LOT NO. 400100**

| ASSAY | ANALYSIS | UNIT | LAB |
|---|---|---|---|
| ***NUTRIENTS*** | | | |
| PROTEIN(PROTEIN ANALYZER) | 21.47 | % | SajoDongA one |
| FAT (ACID HYDROLYSIS) | 5.54 | % | SajoDongA one |
| CALCIUM (EDTA) | 0.81 | % | SajoDongA one |
| PHOSPHORUS(COLORMETRIC) | 0.72 | % | SajoDongA one |
| ASH(FURNACE) | 4.65 | % | SajoDongA one |
| ***HEAVY METALS*** | | | |
| Pb | Not Detected | ppm | KFA |
| Cd | Not Detected | ppm | KFA |
| As | Not Detected | ppm | KFA |
| Se | Not Detected | ppm | KFA |
| Hg | 0.0029 | ppm | KFA |
| Cr | Not Detected | ppm | KFA |
| F | 11.629 | ppm | KFA |
| ***MYCOTOXINS*** | | | |
| Aflatoxin | Not Detected | ppb | KFA |
| Ochratoxin | Not Detected | ppb | KFA |
| ***Microbial Tests*** | | | |
| Salmonella | Not Detected | | KFA |
| ***Carboncompound*** | | | |
| Melamine | Not Detected | ppm | KFA |

*KFA : Korea Feed Association

**This analysis report is issued for the purpose of quality control.**

**PREPARED BY Min, Kyeong Yun** **CONFIRMED BY Ryu, Keun Hyung**

**Table S6.** Diet ingredients of high fat diet group.

| **Product #D12492** | | **gm%** | **kcal%** |
|---|---|---|---|
| Protein | | 26.2 | 20 |
| Carbohydrate | | 26.3 | 20 |
| Fat | | 34.9 | 60 |
| | **Total** | | 100 |
| | **Kcal/gm** | 5.24 | |
| Ingredient | | **gm** | **kcal** |
| Casein, 30 Mesh | | 200 | 800 |
| L-Cystine | | 3 | 12 |
| Corn Starch | | 0 | 0 |
| Maltodextrin 10 | | 125 | 500 |
| Sucrose | | 68.8 | 275.2 |
| Cellulose, BW200 | | 50 | 0 |
| Soybean Oil | | 25 | 225 |
| Lard* | | 245 | 2205 |
| Mineral Mix S10026 | | 10 | 0 |
| DiCalcium Phosphate | | 13 | 0 |
| Calcium Carbonate | | 5.5 | 0 |
| Potassium Citrate, 1 H2O | | 16.5 | 0 |
| Vitamin Mix V10001 | | 10 | 40 |
| Total | | **773.85** | **4057** |
